# Item Selection Methods for Computer Adaptive Testing With Passages

**DOI:** 10.3389/fpsyg.2019.00240

**Published:** 2019-03-05

**Authors:** Lihua Yao

**Affiliations:** Office of People Analytics, Defense Personnel Assessment Center, Defense Human Resource Activity, United States Department of Defense, Seaside, CA, United States

**Keywords:** CAT, item response theory, multidimensional item response theory, MLE, IRT, MIRT, testlet-effect, passages

## Abstract

Computer adaptive testing (CAT) has been shown to shorten the test length and increase the precision of latent trait estimates. Oftentimes, test takers are asked to respond to several items that are related to the same passage. The purpose of this study is to explore three CAT item selection techniques for items of the same passages and to provide recommendations and guidance for item selection methods that yield better latent trait estimates. Using simulation, the study compared three models in CAT item selection with passages: (a) the testlet-effect model (T); (b) the passage model (P); and (c) the unidimensional IRT model (U). For the T model, the bifactor model with testlet-effect or constrained multidimensional IRT model was applied. For each of the three models, three procedures were applied: (a) no item exposure control; (b) item exposure control of rate 0.2 ; and (c) item exposure control of rate 1. It was found that the testlet-effect model performed better than passage or unidimensional models. The P and U models tended to overestimate the precision of the theta or latent trait estimates.

## Introduction

In Reading, Mathematics, Listening, and English tests, students are often asked to respond to several items related to a common stimulus. Information used to answer these items is interrelated in the passage. These kinds of assessments are known to be likely to produce local item dependence (LID). Oftentimes, for simplicity, the related items are scored independently or are summed as one score. Yen (1993) points out two negative measurement effects of ignoring LID in standard item response theory (IRT) parameter estimation and scoring, namely overestimation of the precision of prociency estimates and bias in discrimination parameter estimates.

A testlet can be created by combining the set of inter-related items. For dichotomous scored items, testlet effect has been modeled to include a random testlet effect by modifying the two parameter model (testlet-effect-2PL; Bradlow et al., 1999) and the three-parameter model (testlet-effect-3PL; Waniner et al., 2000), Rasch model (testlet-effect-Rasch; Wang and Wilson, 2005). DeMars (2006) applied the Bi-Factor multidimensional three-parameter logistic (M-3PL) IRT model to the testlet-based test. The testlet-effect-3Pl model is a constrained M-3Pl model in parameter estimation and ability estimation. These models were applied to tests of traditional paper and pencil format.

Computer adaptive testing (CAT) has the advantages of increasing the measurement precision; after each item selection, the examinee's ability is updated and the next selected item will maximally improve the accuracy for this estimated examinee. Testing companies administrate items of passages for content areas such as Reading and Paragraph Comprehension. There are usually two methods in CAT item selection. One method is to use the unidimensional IRT (UIRT) CAT item selection method and treat each item as independent; even though the items in the passage are dependent. The other method is to select the passage with the maximum information or the minimum error variance and to have the items in the passages be all administrated or partially administrated; the information for the passage is the sum of the informations for all the items in the passage.

Murphy et al. (2010) studied and compared the 3PL IRT and 3PL TRT (testlet response theory) item selection methods, and found that they performed similarly. However, the information for the passage is computed based on the sum of item information under the 3PL IRT model, and item dependency is ignored during item selection. Models taking into account the item dependency in item selection for CAT have not been studied.

This study used simulation and compared three models in CAT item selection: (a) the testlet-effect model (T); (b) the passage model(P); and (c) the unidimensional IRT model (U). The newly proposed T model for CAT is compared with two commonly used P and U models. For the T model, the bifactor model (M-3PL) with testlet-effect was applied. The information for each testlet was computed based on M-3Pl model and was used as the item selection criteria. For the P model, the information for the passage was computed based on the sum of item information under the 3PL IRT model, and item dependency was ignored during item selection; this is similar to the TRT model in Murphy et al. (2010). For the U model, the regular 3PL model was applied and the information for each item was used as the item selection criteria.

For each of the three models, three procedures were applied: (a) no item exposure control; (b) item exposure control of rate 0.2 using priority index method; and (c) item exposure control of rate 1 using priority index method. Priority Index (PI) is a method that puts probability or weight on each item in the pool after each item selection.

Various item selection criteria have been proposed and studied. Murphy et al. (2010) has applied MFI (maximum information), MPWI (maximum the posterior weighted information), and MEPV (minimum the expected posterior variance); they found no or little difference among these. For both MPWI and MEPV, integral or weighted summation would require much longer computation time for multidimensional models (T model here). Other methods such as Kullback-Leibler information (Chang and Ying, 1996; Veldkamp and van der Linden, 2002) and Volume (Segall, 1996) methods have been studied and compared in the MIRT frame work (Yao, 2012). The testlet-effect model in this paper was a constrained MIRT model with 39 dimensions. Thus, only the maximum information item selection criteria was applied, as other methods would require much longer computer time.

### Multidimensional Models and TestLet-Effect Models

For a dichotomous scored or multiple choice item *j*, the probability of a correct response to item *j* for an examinee with ability ${\vec{\theta }}_{i}=\left(\theta_{i1},\cdots \;,\theta_{iD}\right)$, following the multidimensional three-parameter logistic (M-3PL; Reckase, 1997, 2009) model is defined as:

(1) $$\begin{array}{r} P_{ij1}=P\left(x_{ij}=1|{\vec{\theta }}_{i},{\vec{\beta }}_{j}\right)=\beta_{3j}+\frac{1-\beta_{3j}}{1+e^{\left(-{\vec{\beta }}_{2j}⊙{\vec{\theta }}_{i}^{T}+\beta_{1j}\right)}}, \end{array}$$

where *x*_*ij*_ = 0 or 1 is the response of examinee *i* to item *j*. ${\vec{\beta }}_{2j}=\left(\beta_{2j1},\cdots \;,\beta_{2jD}\right)$ is a vector of dimension *D* for item discrimination parameters. β_1*j*_ is the scale difficulty parameter. β_3*j*_ is the scale guessing parameter. Here the dot product is defined as ${\vec{\beta }}_{2j}⊙{\vec{\theta }}_{i}^{T}=\overset{D}{\underset{l=1}{\sum }}\beta_{2jl}\theta_{il}$. The parameters for the *j*th item are ${\vec{\beta }}_{j}=\left({\vec{\beta }}_{2j},\beta_{1j},\beta_{3j}\right)$.

For a polytomous scored or a constructed response item *j*, the probability of a response *k*−1 to item *j* for an examinee with ability ${\vec{\theta }}_{i}$ is given by the multi-dimensional version of the partial credit model (M-2PPC; Yao and Schwarz, 2006) :

(2) $$\begin{array}{r} P_{ijk}=P\left(x_{ij}=k-1|{\vec{\theta }}_{i},{\vec{\beta }}_{j}\right)=\frac{e^{\left(k-1\right){\vec{\beta }}_{2j}⊙{\vec{\theta }}_{i}^{T}-\sum_{t=1}^{k}\beta_{\delta_{t}j}}}{\sum_{m=1}^{K_{j}}e^{\left(\left(m-1\right){\vec{\beta }}_{2j}⊙{\vec{\theta }}_{i}^{T}-\sum_{t=1}^{m}\beta_{\delta_{t}j}\right)}}, \end{array}$$

where *x*_*ij*_ = 0, ⋯ , *K*_*j*_−1 is the response of examinee *i* to item *j*. ${\vec{\beta }}_{2j}=\left(\beta_{2j1},\cdots \;,\beta_{2jD}\right)$ is a vector of dimension *D* for item discrimination parameters. β_δ_*k*_*j*_ for *k* = 1, 2, …, *K*_*j*_ are the threshold parameters or Alpha parameters, β_δ_1_*j*_ = 0, and *K*_*j*_ is the number of response categories for the *j*th item. The parameters for the *j*th item are ${\vec{\beta }}_{j}=\left({\vec{\beta }}_{2j},\beta_{\delta_{2}j},\ldots ,\beta_{\delta_{K_{j}}j}\right).$

Testlet-effect-2PPC/3PL model is a constrained M-2PPC/3PL model. This model essentially puts a constraint on the discrimination parameter within each testlet or cluster of inter-related items in a form of a constant. The discrimination parameter varies across testlets to account for the testlet effect. Suppose there are *D*−1 testlets for a test. Then the model can be *D* dimensional IRT model, and the discrimination parameters are

(3) $$\begin{array}{r} {\vec{\beta }}_{2j}=\left(\beta_{2j1},\beta_{2j1}\gamma_{1},\beta_{2j1}\gamma_{2},\cdots \;,\beta_{2j1}\gamma_{D-1}\right) \end{array}$$

where γ = (γ_1_, ⋯ , γ_*D*−1_) are the variances of the testlet-effect parameters for the *D*−1 testlets. Within each testlet, the ratio of the item general discrimination (β_2*j*1_) and the item testlet-effect discrimination is a constant, namely testlet-effect parameter γ_*k*_, where *k* ∈ {1, ⋯ , *D*−1}. The other item parameters (item difficulty/guessing or threshold) remain the same as the general MIRT model. For a testlet-effect model based on a common stimulus, each item belongs to only one testlet, i.e., the discrimination parameters for item *j* is (β_2*j*1_, β_2*j*1_γ_δ_*j*__), where δ_*j*_ ∈ {1, 2, ⋯ , *D*−1}. As in Li et al. (2006) or DeMars (2006), the formulas presented here are consistent with those found in the existing testlet models by Wainer et al. (2007). For item *j* in *kth* testlet-effect,

(4) $$\begin{array}{r} {\vec{\beta }}_{2j}⊙{\vec{\theta }}_{i}^{T}=\beta_{2j1}\theta_{i1}+\beta_{2j1}\gamma_{k}\theta_{ik}, \end{array}$$

and $\gamma_{k}\theta_{ik}\sim N\left(0,\gamma_{k}^{2}\right)$.

Unidimensional model is a special case of the multidimensional model where the dimension D is 1.

## Simulation Study

The models introduced above were used for the simulation study. Item parameters introduced are unknown at the beginning; generally, a try out or a field test is conducted to collect responses from test-takers and the item parameters would then be estimated by an existing software. For CAT, item parameters in the pool should be known. Two set of item parameters are derived as described below using the real responses from test-takers taking paper and pencil format test.

### Item Pool

Two sets of item pools were derived from BMIRT (Yao, 2003), based on real data listening assessments; there were 100 items with 38 testlets and 16 single items with 5000 examinees. Most of the testlets had two items, with some having 3 or 4 items. There were 16 items that were independent from each other and from others. The testlet-effect model of 39 dimensions and the unidimensional IRT models were applied to the data. The 38 estimated testlet-effect parameters varied from 0.05 to 0.7. Item Pool One had 100 testlte-effect item parameters. Item Pool Two had 100 UIRT item parameters. The two models fit the data equally well. The correlations between the estimates for the two models for the discrimination, difficulty, and guessing parameters were 0.97, 0.99, and 0.91, respectively; the first discrimination was used for the testlet-effect model. The AICs were 501917 and 527192 for the UIRT model and the testlet model, respectively. The chi-square difference between the two models was 1.78, which was considered as not significant.

Pool One had 100 items with item parameters of 39 dimensions following the testlet-effect model and was used for T model selection method. Pool Two had 100 items with item parameters of the unidimensional 3PL model and was used for U model selection. Pool Two was also used for the P model selection method.

### The Priority Index for Item Exposure Control

The multidimensional priority index (MPI, Cheng and Chang, 2009; Yao, 2013) for each item *j* is defined by

(5) $$\begin{array}{r} MPI_{j}=\overset{D}{\underset{l=1}{\prod }}f_{jl}^{c_{jl}}, \end{array}$$

where the constraint matrix *C* = (*c*_*jl*_)_*J*×*D*_, indicating the loading information for item *j* on domain *l*, is defined as the following:

$$\begin{array}{l} c_{jl}=\left\{\begin{array}{cc} 1 & \text{if item j load on domain l}\\ 0 & \text{otherwise} \end{array}\right\} \end{array}$$

For the *j*th item, let *r*_*j*_ denote its exposure rate. For each selection step, let *n*_*j*_ be the number of examinees that have already selected item *j*. The index for the item exposure control is defined by (van der Linden and Veldkamp, 2004, 2007; Yao, 2013)

(6) $$\begin{array}{r} f_{jl}=\max\left\{\frac{r_{j}-\frac{n_{j}}{N}}{r_{j}},0\right\}, \end{array}$$

where *N* is the total number of examinees. This index will make sure that no item is selected with exposure rate larger than the predefined rate $\vec{r}=\left(r_{1},\cdots \;,r_{J}\right)$. At the beginning of item selection, the weight or the probability of being selected for the item is high. If the item is administrated, then the weight or the probability for the item is smaller, until it reaches 0, then this item will not be selected anymore.

For the testlet-effect model and the passage selection model, there were only 54(38 + 16) passages or items in the pool. Thus, for 2,000 examinees, item exposure rate of 0.2 would result in no items being selected if all the item priority index were 0 after reaching exposure rate. Therefore, a modification was made after all items had reached exposure rate; the probability was reset for each item in the pool; although a higher exposure rate could be applied to avoid this problem.

Three methods were used for item exposure control in this study: no item exposure control and exposure rate of 0.2 and 1 using the Priority Index.

### True Abilities

For this simulation, 2,000 examinees (true abilities) were sampled from the standard multivariate normal distribution of dimension 39; these examinees were used for the T model. For the P and the U model, the first/general ability was used.

### CAT Item Selection Methods

For all item selection methods, the first item or passage was randomly selected from the top 20, and the second item or passage was randomly selected from the top 10. There were three types of models for the item selection methods: the testlet-effect model, the unidimensional passage based model, and the unidimensional IRT based model. Maximum information was used as the criteria for all methods. MAP ability estimates were used to update ability after each item or passage selection. Testlet lengths of 10, 20, and 30 were specified. For the testlet-effect model and the passage selection models, if all items in the selected testlet or passage were administered, then the actual number of items selected for each examine might have been slightly higher than 10, 20 or 30, because the last selected passage may have had multiple items. Therefore, three methods for selecting items with passages were proposed in this study (for both T and P methods): (1) M1: select all items in the passage until the fixed test length is reached; (2) M2: select all items in the selected passages except the last selected passage—the items in the passage are partially or all selected until the fixed test length is reached; (3) M3: select partial items in the passage and stop the selection if the fixed test length is reached. So for method M1, the test length might have been longer than the fixed test length, but methods M2 and M3 had the fixed test length.

The item selection procedures are briefly described below. For all the procedures, the initial abilities are set to θ_*l*_ = 0 for *l* = 1, ⋯ , *D*. For *j* = 2, ⋯ , *J*, suppose *j*−1 items have been selected. To select the next *j*th item, suppose the updated ability is ${\vec{\theta }}^{j-1}$. For each of the procedures, the steps proposed are repeated. Please note that the procedures are for both Bayesian and non-Bayesian; for Bayesian, add Σ^−1^ to the information.

### Steps for the T Methods

1. For each passage *M*_*p*_ in the pool (including single items), compute the information at the ability level ${\vec{\theta }}^{j-1}$,
  $$\begin{array}{l} {\text{I}}_{M_{p}}\left({\vec{\theta }}^{j-1}\right)=\underset{m\in M_{p}}{\sum }\frac{{\left(P_{m1}-\beta_{3m}\right)}^{2}\left(1-P_{m1}\right)}{P_{m1}{\left(1-\beta_{mj}\right)}^{2}}{\vec{\beta }}_{2m}⊗{\vec{\beta }}_{2m} \end{array}$$
  where *m* ∈ *M*_*p*_ is for all the items in the testlet or passage *M*_*p*_.
2. Select the passage *M*_*p*_ such that **I**_*M*_*p*__[0][0] has a maximum value (among all the passages in the pool); it is the element in the top right of the information matrix and it measures the precision of the primary ability.
3. Rank order the items in the passage *M*_*p*_ by their informations and select the top *n*_*p*_ items where *n*_*p*_ = *m*_*p*_ or *n*_*p*_ = *Math*.*min*(3, *m*_*p*_/2 + 1) for selection method M1 and M3 respectively, where *m*_*p*_ is the number of items in passage *M*_*p*_. For selection method M2, *n*_*p*_ = *m*_*p*_ for all except the last selected passage. The process stop if the test length reached the maximum for method M2 and M3.
4. Update ability ${\vec{\theta }}^{j}$ based on the selected *j*−1+*n*_*p*_ items.

#### Steps for the P Methods

For the P method, unidimensional IRT is used. The above steps were similarly applied; the number of dimensions is 1.

#### Steps for the U Methods

U model is a special case when the number of dimensions is 1; the information function is a scalar. The steps are below:

1. For each item *m* in the pool, compute the information the ability level ${\vec{\theta }}^{j-1}$,
  $$\begin{array}{l} {\text{I}}_{j}^{m}\left({\vec{\theta }}^{j-1}\right)=\frac{{\left(P_{m1}-\beta_{3m}\right)}^{2}\left(1-P_{m1}\right)}{P_{m1}{\left(1-\beta_{mj}\right)}^{2}}{\vec{\beta }}_{2m}⊗{\vec{\beta }}_{2m} \end{array}$$
2. Select item *j* = *m* such that ${\text{I}}_{j}^{m}\left({\vec{\theta }}^{j-1}\right)$ has a maximum value (among all the items in the pool).
3. Update ability ${\vec{\theta }}^{j}$ based on the selected *j* items.

For ability update and final estimates, there are five basic estimation methods that are used in IRT: (a) maximum likelihood estimation (MLE; Lord, 1980, 1984), (b) Maximum a posterior (MAP; Samejima, 1980), (c) Owen's sequential Bayesian (OSB; Owen, 1975), (d) expected a-posteriori (EAP; Bock, 1981), (e) marginal maximum likelihood (MML; Bock, 1981), and (f) weighted maximum likelihood (Warm, 1989). Owen method has been widely used in the process of updating ability after each item selection in unidimensional CAT; the final ability estimate is still non Owen method such as MAP or MLE; the item order affects Owen estimates. Owen method in updating ability is fast and accurate, as it uses only the response for the current selected item and previous ability update. The extension of Owen method in updating the vector ability to the MIRT model would be so much faster than any other methods. However, no research has been conducted.

Studies have been conducted comparing the performance of MAP, weighted MLE, and MLE in unidimensional IRT (Wang and Wang, 2001; Penfield and Bergeron, 2005; Sun et al., 2012). The comparison was extended to the MIRT model in Yao (2018); it was for a fixed length paper and pencil test. It was found that Bayesian method MAP had larger BIAS but smaller SE (standard error) and RMSE. WMLE and MLE had similar results with WMLE performing slightly better than MLE; both had smaller BIAS but larger RMSE and SE compared to MAP. WMLE used the largest computer time in estimating ability and MAP used the smallest computing time. For the simulated data in this research, WMLE for ability update and estimates would require much longer computer time. Therefore, MAP estimation method was used.

### Evaluation Criteria

Reliability, BIAS, and SE (standard error) for the abilities were computed average over replications. They were defined as follows: let *f*_*true*_ be the value of a function obtained from true parameter and *f*_*l*_ be the value of a function obtained from the estimated parameter from sample *l*. Here the function *f* can represent ability parameters. RMSE was calculated by $RMSE=\sqrt{\frac{1}{n}\overset{n}{\underset{l=1}{\sum }}{\left(f_{l}-f_{true}\right)}^{2}}$, where *n* is the number of replications. BIAS was defined by $BIAS=|f_{true}-\bar{f}|$, where $\bar{f}=\frac{1}{n}\overset{n}{\underset{l=1}{\sum }}f_{l}$ was the final estimate. Standard error SE was calculated by $SE=\sqrt{\frac{1}{n}\overset{n}{\underset{l=1}{\sum }}{\left(f_{l}-\bar{f}\right)}^{2}}$. Standard error of measurement for content domain *l* was calculated by $SEM_{l}=\sqrt{{\vec{\alpha }}_{l}{\text{I}}^{-1}{\vec{\alpha }}_{l}^{T}}$, where ${\vec{\alpha }}_{l}=\left(0,\cdots \;,1,0,\cdots \;\right)$ has 1 in the *l*th element. For Bayesian method, replace **I**^−1^ with (**I** + Σ^−1^)^−1^. Here **I** is the information for the test at the estimated abilities. The reliability is the square of the correlations between the estimates and the true values.

Item usage and test overlap rate were computed to evaluate the item pool usage. Test overlap rate is the expected number of common items encountered by two randomly selected examinees divided by the expected test length. It can be derived below:

(7) $$\begin{array}{r} Overlap=\frac{\sum_{j=1}^{T}count_{j}\left(count_{j}-1\right)}{J\left(N\left(N-1\right)\right)}. \end{array}$$

If the exposure rate of an item is bigger than 0.2, then it is defined as overexposed. If the exposure rate of an item is smaller than 0.02, then it is defined as underexposed.

## Results

**Figure 1:**
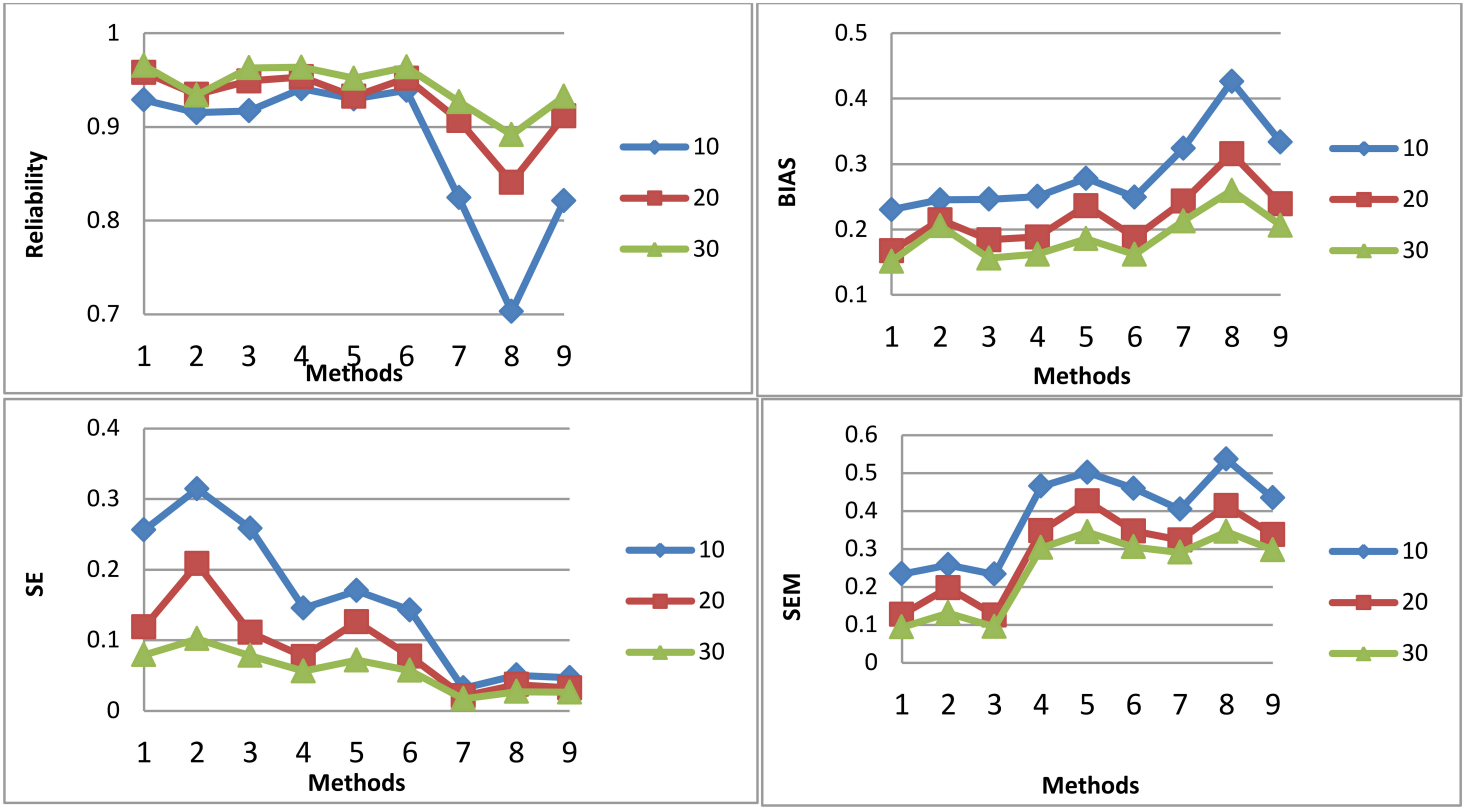
Reliability, BIAS, empirical SE, and SEM for test length of 10, 20, and 30 for all 9 procedures for selection method M1.

**Figure 2:**
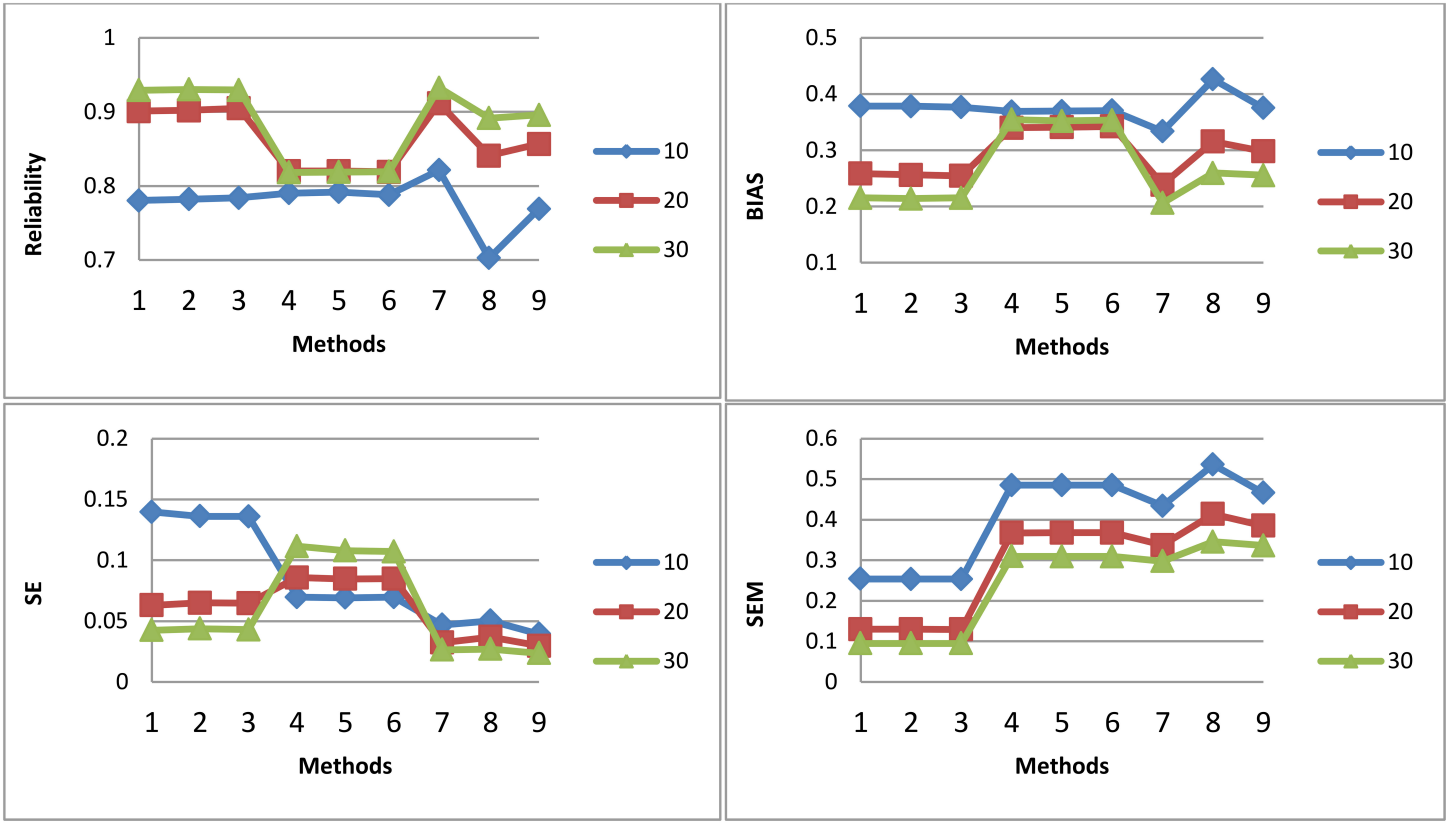
Reliability, BIAS, empirical SE, and SEM for test length of 10, 20, and 30 for all 9 procedures for selection method M2.

**Figure 3:**
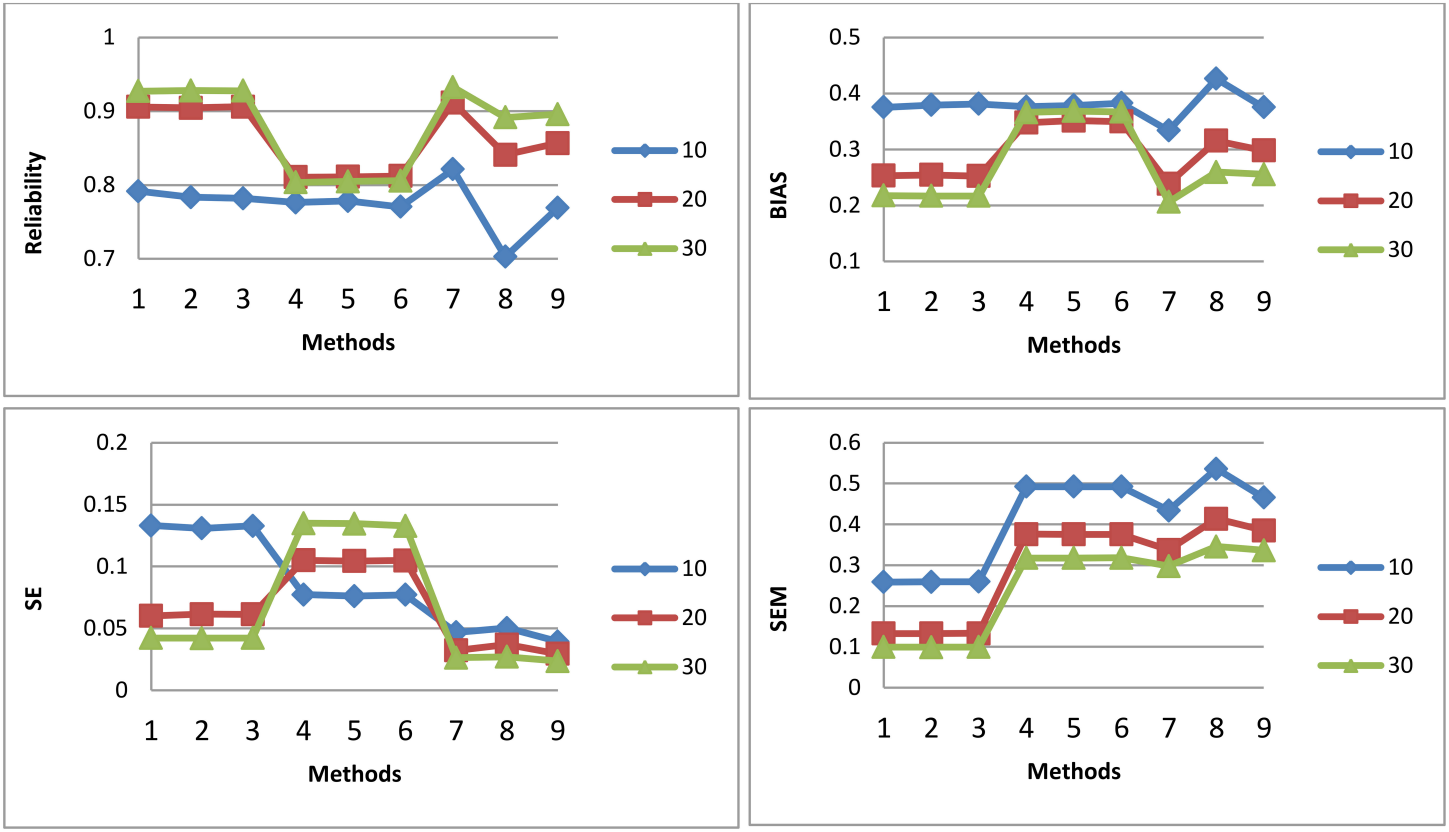
Reliability, BIAS, empirical SE, and SEM for test length of 10, 20, and 30 for all 9 procedures for selection method M3: Note about the x-axis for the 9 procedures: 1, Testlet model; 2, Testlet model with 0.2 as item exposure rate using PI; 3, Testlet model with 1.0 as item exposure rate using PI; 4, Passage model; 5, Passage model with 0.2 as item exposure rate using PI; 6, Passage model with 1.0 as item exposure rate using PI; 7, UIRT model; 8, UIRT model with 0.2 as item exposure rate using PI; 9, UIRT model with 1.0 as item exposure rate using PI.

Figures 1–3 shows the reliability, BIAS, empirical SE, and SEM for test length of 10, 20, and 30 for all 9 procedures for the general ability (the first dimension) for methods M1-M3, respectively. The U methods had the smallest SE; the standard errors under the IRT model were overly optimistic. This finding is consistent with previous research (Yen, 1984; Sireci et al., 1991; Wainer and Thissen, 1996; Wainer et al., 2007; Murphy et al., 2010). The U models tend to overestimate the precision of the theta estimates when the dependency of items is ignored. For M1, the T model is the best in reliability, BIAS, and SEM; the T and P models may have longer test length than U for M1. However, for M2 and M3, T and P had the same test fixed test length as U. For both M2 and M3, the T model also had the best reliability, BIAS, and SEM. The P model had the smallest reliability and larger BIAS and SEM compared to the T; the dependency of items for the P model was ignored. The P model performed the worst. For the T model, compared to M2, M3 had slightly higher reliability and smaller SE, although the difference was not significant. For M2 and M3, the results for the T and P for the three item exposure control were similar; this was not surprising, as there were only 38 passages and 16 single items in the pool to be selected. For the U model, the item exposure control had an effect, as there were 100 items to be selected in the pool.

**Figure 4:**
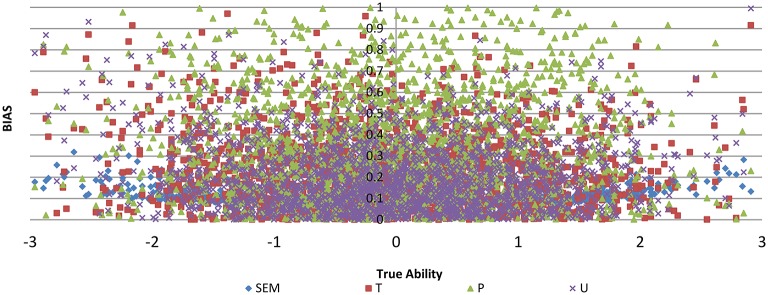
BIAS against true ability for three item selection methods T, P, and U, with no item exposure control for test length of 30 for method M2.

Figure 4 shows the BIAS against true ability for the three item selection methods T, P, and U, with no item exposure control for test length of 30 for M2. It can be seen that T had smaller BIAS for most of the abilities than the P and U models. To see the bias in detail, the 2,000 true abilities were classified into four categories with their means for each category computed; the means for the true ability, BIAS and SEM for each category were computed. Table 1 displays the results. For all four categories, the T model had the smallest BIAS and SEM. Table 2 shows the percentage of misclassification rate for the three models T, P, and U. The second column is the category based on the true ability values and the last column is the percentage of misclassification based on the estimated values from the models; for the four cut points in Table 1, there were 5 categories. The three models had similar misclassification rates for categories 2, 3, and 4, but there were differences for categories 1 and 5, with T performing the best. Please also note that the T model had the smallest SEM (standard error of measurement).

**Table 1:** True ability, BIAS, and SEM for T, P, and U methods for test length of 30 with no item exposure control.

**Methods**	**Category**	**True Ability**	**BIAS**	**SEM**
T	1	−1.3088	0.2398	0.1058
P	1	−1.3088	0.3859	0.3173
U	1	−1.3088	0.2351	0.3097
T	2	−0.3321	0.2174	0.0978
P	2	−0.3321	0.3338	0.3182
U	2	−0.3321	0.1908	0.2992
T	3	0.3089	0.2040	0.0926
P	3	0.3089	0.3437	0.3169
U	3	0.3089	0.1837	0.2921
T	4	1.2652	0.2098	0.1019
P	4	1.2652	0.4016	0.3185
U	4	1.2652	0.2154	0.2897

**Table 2:** Misclassification rate for T, P, and U methods for test length of 30 with no item exposure control.

**Methods**	**Category**	**Percentage**
T	1	2.8
P	1	3.7
U	1	3
T	2	24.55
P	2	24.45
U	2	25.15
T	3	25.25
P	3	25.25
U	3	25.25
T	4	27.55
P	4	27.55
U	4	27.55
T	5	1.1
P	5	2.4
U	5	2.6

**Figure 5:**
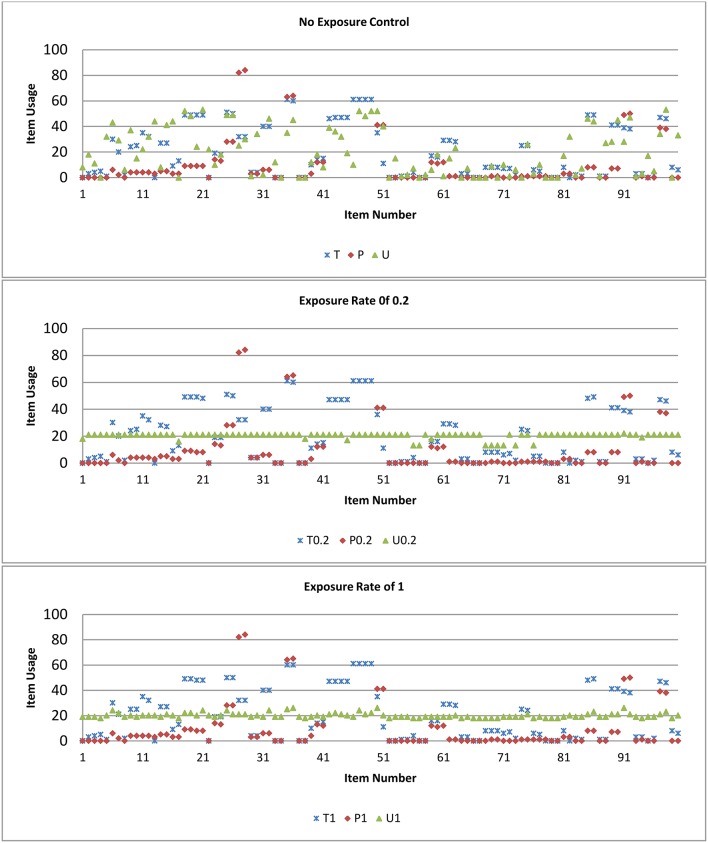
Item usage rate for the three methods T, P, and U for conditions of item exposure rate of 0.2, 1, and no exposure control for test length of 30 for selection method M2.

Figure 5 plots the item usage rate for the 100 items (x-axis) for the three methods T, P, and U for conditions of item exposure rate of 0.2 and 1, and of no exposure control for M2. For item exposure rate of 0.2, all items were being selected and the maximum exposure rate for items was under 30% for the U model. There were some items that were never selected including testlets with 2 or 3 items within; the maximum exposure rate for items was under 60%, and 85% for the T and P models, respectively. Passage with 4 items within had a higher selected rate, although there were some passages with 3 items within that had a smaller usage rate. For the P model, there were more items, compared to the T and U models, that had 0 or small item usage.

**Figure 6:**
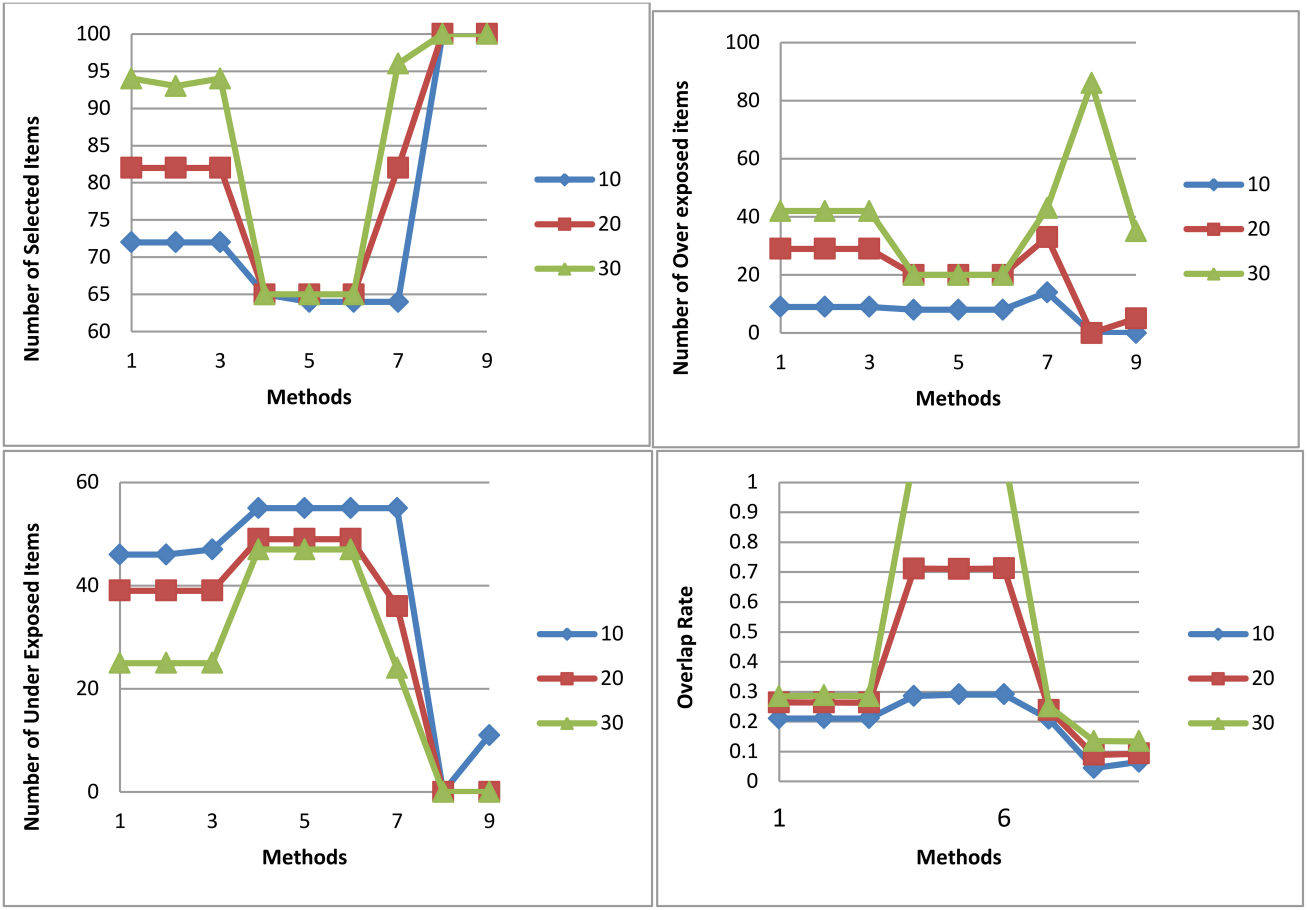
Number of selected items, number of over exposed items, number of under exposed items, and Item overlap rate for all three test lengths and 9 procedures for selection method M2.

Figure 6 shows the number of selected items, the number of over exposed items, the number of under exposed items, and the item overlap rate for all three test lengths and 9 procedures for M2. Over exposed items were those that had an exposure rate higher of than 0.2; under exposed items were those that had an exposure rate of smaller than 0.02. Overall, the P model had a narrow range of items that were selected. The U model had the smallest overlap rate followed closely by the T model. The T model had a similar item overlap rate as the U model when there was no item exposure control. For test length of 20, the T model and the U model with no item exposure control had similar item usage. Therefore, it is expected that with larger number of testlet items in a pool, the T model should have as good usage of items as the U model.

## Discussion

This simulation study compared three models in CAT item selection with passages: (a) the testlet-effect model (T); (b) the passage model(P); and (c) the unidimensional IRT model (U). It was found that TRT (P) and IRT (U) performed similarly in Murphy et al. (2010); however, in this study, the T and P methods were more closer to each other than to the U in reliability and BIAS; the P method here was the same method as the TRT. Both P and U methods tended to overestimate the precision of the theta estimates, as the dependency of items was ignored; both had smaller SE, larger BIAS and SEM. The T model, where the testlet-effect was considered, had the best reliability and the smallest BIAS and SEM. Moreover, the item pool usage for T models could be as good as the U models when there were more testlet items in a pool. Specific or a required number of single items could be selected; this would be helpful when there are not many testlet items. Even though 0 number of required single items were specified in this study, the software can be adopted to handle any input number and is free for the public to use.

Real data was used in deriving the item pools for the models. The UIRT model and the testlet-effect model fit the real data equally well so the comparison between different CAT item selection methods from different models make sense. In real practice, if the UIRT model fits the model the best, then the testlet-effect is not strong, and the UIRT model should be used. Many testing companies still use the U model—for example, the CAT ASVAB military test for content PC(Paragraph Comprehension). The U model is studied here as the baseline. If the testlet-effect model fits the data the best, then it is expected that the testlet-effect model for CAT(T) would be much better than the UIRT model (U) or the passage (P) model. Future study varying the degree of testlet-effect and comparing the performance of T and U and other methods should be conducted; for example, treating items in a testlet as a polytomously scored items modeled by the multidimensional generalized two-parameter partial credit model (Yao and Schwarz, 2006).

For this study, the number of passages in the pool was small, therefore, different item exposure controls for both T and P yielded similar results. For pools with larger numbers of passages, the relative performances of T, P, and U would be similar to this study in the case where there is no item exposure control. Overall, the T model is recommended.

Fixed length test was used in this study for the U model and also for the T and P models for M2 and M3. For the passage selection method, partial items or all items in the pool could be selected. Three methods (M1, M2, and M3) were proposed for the T model; other selection criteria can be studied.

This research used only maximum information method (MFI) in selecting items. Other methods such as minimum expected posterior variance (van der Linden and Pashley, 2000), Kullback-leibler information (Chang and Ying, 1996), and Volume (Segall, 1996) methods could be studied and compared for T, P, and U models. Similar observations should be expected; however, they might require longer computer time. For CAT using the testlet-effect model, the number of dimensions is the number of testlet plus 1. Therefore, if the number of testlet or cluster in the item pool is larger, then the number of dimensions is larger; other item selection methods besides MFI would be impossible. If the number of testlet or cluster in the item pool is small, then the number of dimensions is small. Most of the items in the pool would be single items. Thus, the relative performance of item selection criteria would be similar to the results as in Yao (2012, 2013).

Other models that consider testlet-effect and content domain information simultaneously can be proposed and studied. Suppose there are *K* testlets and *D*+1 content domains, with the first dimension as the general dimension measuring general ability, and the rest of the D dimensions are content specific dimensions. Then the model can be *D*+1 dimensional IRT model, and the discrimination parameters are

(8) $$\begin{array}{r} {\vec{\beta }}_{2j}=\left(\beta_{2j1},\beta_{2j1}\gamma_{1},\beta_{2j1}\gamma_{2},\cdots \;,\beta_{2j1}\gamma_{K}\right) \end{array}$$

where γ_1_, ⋯ , γ_*K*_ are testlet-effect parameters for the *K* testlet. Within each testlet, the ratio of the item general discrimination (β_2*j*1_) and the item testlet-effect discriminations is a constant, namely testlet-effect parameter γ_*k*_, where *k* ∈ {1, ⋯ , *K*}. The other item parameters (item difficulty/guessing or alphas) remain the same as the general MIRT model. The number of dimensions and the number of testlets can be different. When the number of dimensions is the number of testlets+1, then it is the regular testlet model that was studied in this paper.

## Author Contributions

The author confirms being the sole contributor of this work and has approved it for publication.

### Conflict of Interest Statement

The author declares that the research was conducted in the absence of any commercial or financial relationships that could be construed as a potential conflict of interest.
